# Transfer Learning-Based Condition Monitoring of Single Point Cutting Tool

**DOI:** 10.1155/2022/3205960

**Published:** 2022-07-15

**Authors:** S. Naveen Venkatesh, P. Arun Balaji, M. Elangovan, K. Annamalai, V. Indira, V. Sugumaran, Vetri Selvi Mahamuni

**Affiliations:** ^1^School of Mechanical Engineering, VIT University Chennai Campus, Vandalur-Kelambakkam Road, Keelakottatiyur, Chennai 600127, India; ^2^Department of Mechanical Engineering, SNS College of Technology, Coimbatore, India; ^3^Department of Mathematics, Indira Gandhi College of Arts and Science, Kathirkamam, Puducherry, India; ^4^Department of Project Management, Mettu University, P.O. Box 318, Metu, Ethiopia

## Abstract

Machining activities in recent times have shifted their focus towards tool life and tool wear. Cutting tools have been utilized on a daily basis and play a vital role in manufacturing industries. Prolonged and incessant operation of the cutting tool can lead to wear and tear of the component, thereby compromising the dimensional accuracy. The condition of a tool is estimated based upon the surface quality of the machined component, condition of the machine, and the rate of production. Maintaining the tool health plays a vital role in enhancing the productivity of manufacturing industries. Numerous efforts were experimented by the researchers to maintain the tool health condition. The drawbacks of conventional diagnostic techniques include requirement of high level of human intelligence and professional expertise on the field, which led the researchers to develop intelligent and automatic diagnostic tools. There are many techniques suggested by researchers to detect the condition of single point cutting tool. This article proposes the use of transfer learning technology to detect the condition of single point cutting tool. First, the vibration signals were collected from the cutting tool and plots were made which will work as input to the deep learning algorithms. The deep learning algorithms have the capability to learn from the plots of vibration signals and classify the state of the single point cutting tool. In this work, the pretrained networks such as VGG-16, AlexNet, ResNet-50, and GoogLeNet were employed to identify the state of the cutting tool. In the pretrained networks, the effect of hyperparameters such as batch size, solver, learning rate, and train-test split ratio was studied, and the best performing network was suggested for tool condition monitoring.

## 1. Introduction

In the present high-speed machining era, continual monitoring of the tool condition is considered vital such that enhanced surface finish is achieved along with uninterrupted productivity. Workpiece and cutting tool damages can be prevented through early diagnosis of the tool condition with the help of expert systems. Stipulated order realization dates and critical deadlines have urged the necessity for tool condition monitoring such that uninterrupted production is assured to sustain among growing competitions. Detecting faults at an early stage can help in reducing downtime and loss of productivity. Also, corrective preventive maintenance can be scheduled to avoid more severe damages to the machinery and components. Sudden breakdown of machinery or tool can elevate the cost of maintenance and downtime [1, 2]. The aforementioned scenarios created a buzz among industrial practitioners and academic researchers to look into more modern and highly efficient condition monitoring techniques. The process of tool condition monitoring is composed of the collection, processing, and analysis of data acquired during experimentation involving tool condition, and the results are interpreted with respect to the real-time application.

Tool condition monitoring is carried out in several phases comprising of various techniques carried out in a sequential manner. Selection of optimal parameters to be captured, extraction of features, selection of features, and classification of features are the phases included during tool condition monitoring. Several research works have been carried out since the 1980s towards the field of condition monitoring that are robust, effective, and computationally simple. Kannatey-Asibu and Emel performed a linear discriminant function analysis on a titanium carbide-coated cutting tool using acoustic emission resulting in 90% tool breakage [3]. Silva et al. diagnosed the tool condition with the help of adaptive resonance theory (ART) and self-organizing maps using Taylor's tool life equation. The features were extracted from frequency spectrum and multiple sensor statistical transformations [4]. Scheffer and Heyns monitored tool wear during turning operation using strain and vibration measurement acquired simultaneously. Self-organizing maps were adopted to classify features extracted from time domain, frequency domain, wavelet packet analysis, and coefficient of time-series model achieving a near 100% classification accuracy. The correlation coefficient approach was used to select the most significant features [5]. Sick carried out a review on online and indirect tool condition monitoring techniques in turning operation [6]. Several techniques including artificial neural networks (ANN) along with their methodologies were compared and presented. Scheffer and Heyns performed tool condition monitoring in industrial turning operation of aluminium alloys using vibration signals and neural networks [7]. A feed forward back propagation network was used by Alonso and Salgado to evaluate the tool flank wear with the aid of vibration signals and singular spectrum analysis [8]. Dutta et al. adopted texture analyses on machined surface images to predict tool wear using support vector regression. Grey level co-occurrence matrix, discrete wavelet transform, and Voronoi tessellation were used for image texture analysis [9]. Technological advancements in condition monitoring have insisted in going towards many renowned techniques like infrared thermography. However, such processes are quite cost-consuming and require skilled personnel [10]. Based on the literatures discussed above, one can infer that vibration signals were predominantly used in the field of condition monitoring. However, signal processing of vibration signals is challenging due to their dynamic nature and complexity.

Several features like histogram, statistical, autoregressive moving average, and wavelet were adopted by many researchers to monitor the condition of the tool [11]. Additionally, various feature selection techniques like principal component analysis and decision trees were utilized along with the aforementioned features [12]. The classification of the selected features was performed using numerous families of machine learning algorithms like Bayes family, Trees family, Lazy family, and Meta family. Although several research works involving the usage of machine learning algorithms for tool condition monitoring were reported in literature, the effectiveness and efficiency of machine learning highly depends upon the quality of features extracted and selected. In the present scenario, feature engineering proves to be a challenging task due to the complexity present in vibration signals. Considering the abovementioned challenges, researchers have shifted their focus towards developing an automated technology that is capable of classifying instances without extracting explicit features from raw signals. In recent times, deep learning is found to be an effective solution to solve condition monitoring problems by learning image features to provide accurate classification. The image features are learnt automatically within the convolutional neural networks (CNNs), and classification is performed without any separate feature extraction, selection, and classification tool. Deep learning techniques are aimed at working with images. Image acquisition during tool operation is considered challenging, unsafe, and a costly affair.

Vibration signal acquisition from the tool holder is considered as an established activity. The acquired vibration signals can be plotted and used as signature values to detect the condition of the tool. Deep learning techniques (convolutional neural networks) are capable of learning image features from the input data and performing classification according to the predefined classes. This study is a novel attempt to verify the effectiveness of deep learning techniques with vibration plots in detecting the condition of the tool. Renowned and well-established pretrained deep learning models such as AlexNet [13], VGG-16 [14], GoogLeNet [15], and ResNet-50 [16] were considered in the study to perform tool condition monitoring. The aforementioned network models are termed pretrained since these networks were trained on several established datasets consisting of numerous classes and objects. Such pretrained networks have prior knowledge (due to training) that can be transferred and used for solving custom problems (knowledge transfer to different domain). This process of knowledge transfer is termed as transfer learning which is a subset of deep learning [17]. In this study, vibration plots are adopted instead of real-time images due to certain constraints that are listed as follows: (i) acquiring images requires a camera to be placed near the tool holder which is considered as a challenging task, (ii) the image acquisition can be hindered by the coolant supply during turning operation, and (iii) under real-time operation, the machine vibrations can disturb the image quality [18]. In recent times, advances in algorithm have been developed to elevate the level of classification accuracy. An iterative learning control was developed by Zhuang et al., to solve tacking error by randomly varying the trial lengths using successive projection [19]. In another study, an adaptive dynamic programming was developed to control a hydraulic servo actuator [20]. A reinforced learning model was adopted to solve multiplayer non-zero-sum games. The proposed technique used Markov jump linear systems [21].

Recent developments in artificial intelligence (AI) and Internet of things (IoT) have instrumented the development towards “Industry 4.0.” AI is considered as a powerful tool in the field of big data processing and data exploration. Modern intelligent fault diagnosis techniques are built on theories and concepts based on AI. Hinton et al. first proposed deep learning, an AI strategy, in the field of science, which initiated the wave of research into different fields of study [22, 23]. A deep learning strategy (also termed as deep neural networks) consists of a number of neural layers stacked in a hierarchical structure that extracts information from the input. The architecture is called “deep” since the raw data information is learnt through multiple levels of layer-by-layer procedure [24]. Starting from the raw data input, a deep neural network (DNN) discovers the structure of complicated datasets and automatically learns the most significant features through several layers. Automatic feature learning capability and improved nonlinear regression ability have made deep learning models to be widely used in language processing, object detection, visual inspections, surveillance, and robotics. Hence, there is a critical need and great potential to utilize the automatic learning capability of DNN in the field of mechanical systems fault diagnosis.

**Figure 1:**
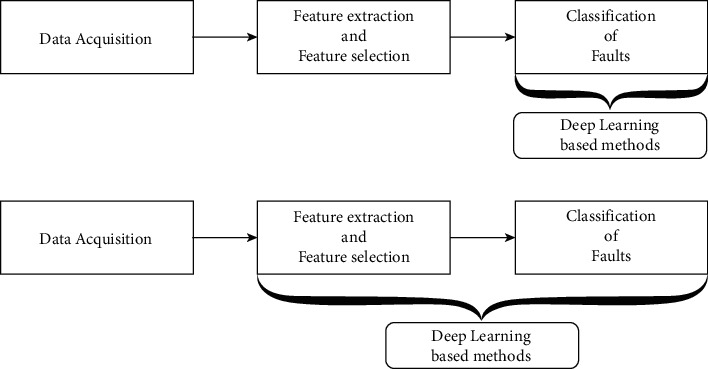
Stages of deep learning application in mechanical systems.

In the past few years, DNN was adopted by many researchers to either perform classification or feature selection in advanced fault diagnosis imitating conventional techniques. Initially, the features from acquired signals were extracted using various feature extraction techniques that are utilized to classify DNN models. Several authors reported on the abovementioned strategy that is discussed as follows: A deep belief network was proposed by Li et al. to diagnose the condition of bearings and gearboxes using statistical features obtained from frequency, time, and time-frequency domain [25]. Chen et al. adopted a convolutional neural network (CNN) to detect the condition of the gearbox using frequency and time features [26]. An air compressor fault diagnosis was carried out by Verma et al., using a sparse autoencoder [27]. Shao et al. used an optimized deep belief network to diagnose bearing faults using 18-time features [28]. In this stage of fault diagnosis, DNN was used only as a replacement for classifier and the prime advantage of DNN feature learning was not equipped completely (Figure 1(a)). After 2015, researchers started using DNN for feature learning and feature selection along with classification. This provided a complete suite for all the activities in one go. Here, DNNs have taken images as input and corresponding classes as output. The DNNs are learnt without explicit need for feature extraction and feature selection. The literatures based on the abovementioned stage are discussed as follows: Condition monitoring of roller bearings was carried out by Guo et al., using deep CNN consisting of two ensembles. Feature extraction and fault pattern recognition were carried out in one CNN, while the fault classification was performed in the other CNN [29]. Zhao et al. performed tool condition monitoring using a convolutional long short-term memory (C-LSTM) [30]. Owing to the feature learning capability of DNN, the process of intelligent fault diagnosis grows into an effective and more automated approach (Figure 1(b)).

CNN forms the basic blocks of deep learning that have been adopted to learn complex features from image data. CNN is considered as one of the most dominant approaches in fault diagnosis, object detection, and speech recognition. Additionally, the application of CNN in tool condition monitoring was not attempted. Some related works representing the application of CNN in mechanical systems are presented in Table 1. In this study, the performance of various state-of-the-art pretrained networks like VGG-16 [14], AlexNet [13], ResNet-50 [16], and GoogLeNet [15] was evaluated for detecting the tool condition from images acquired from vibration signals. Experiments were carried out on the pretrained networks by varying the train-test split ratio and various hyperparameters such as batch size, learning rate, and solver. The derived results were compared and tabulated to identify the suitable network for condition monitoring of the carbide tool. The technical contributions in this study are provided as follows:

- This study considers four tool conditions, namely tool tip unused and good condition (good), tool low blunt (tblent1), tool high blunt (tblent2), and tool tip loose (tiploose).
- Vibration signals were obtained from the tool holder and the plots of vibration signals were utilized as input for pretrained networks.
- Four pretrained networks, namely VGG-16, AlexNet, ResNet-50, and GoogLeNet were considered in the study to perform condition monitoring of the tool.
- Various hyperparameters such as batch size, solver, learning rate, and train-test split ratio were altered, and the performance of the pretrained networks was assessed.
- The best performing network for condition monitoring of the tool was identified based on the results obtained.

## 2. Experimental Studies

**Figure 2:**
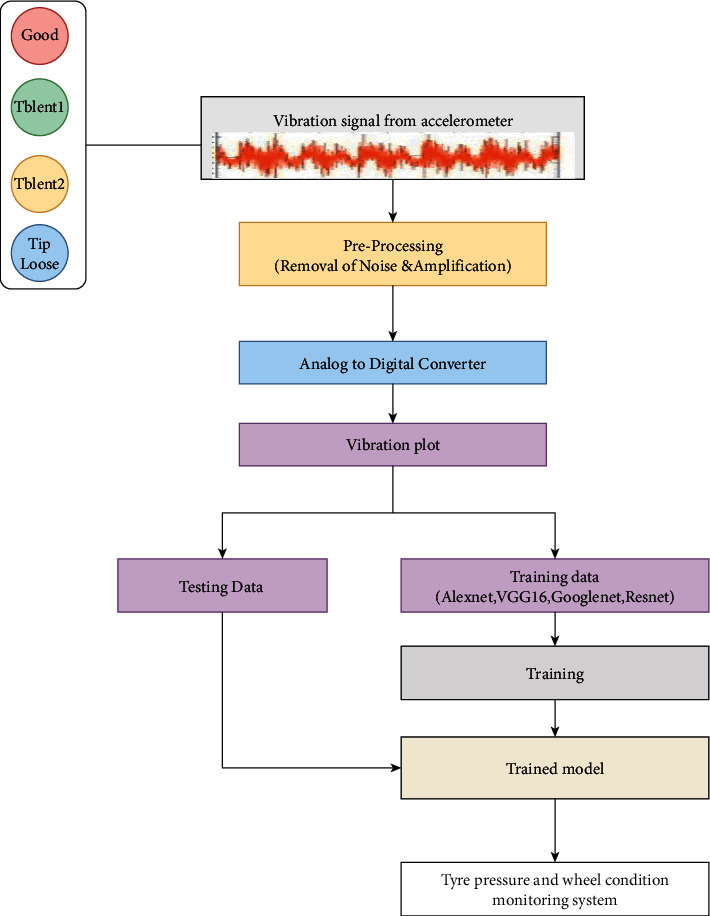
Overall methodology of condition monitoring in tool.

The experimental study is discussed in this section in four folds, namely, experimental setup, data acquisition, fault simulation, and experimental procedure. The experimental setup comprises a single point cutting tool equipped in a CNC turning machine. The complete methodology involved in the process of condition monitoring of the tool is depicted in Figure 2.

### 2.1. Experimental Setup

**Figure 3:**
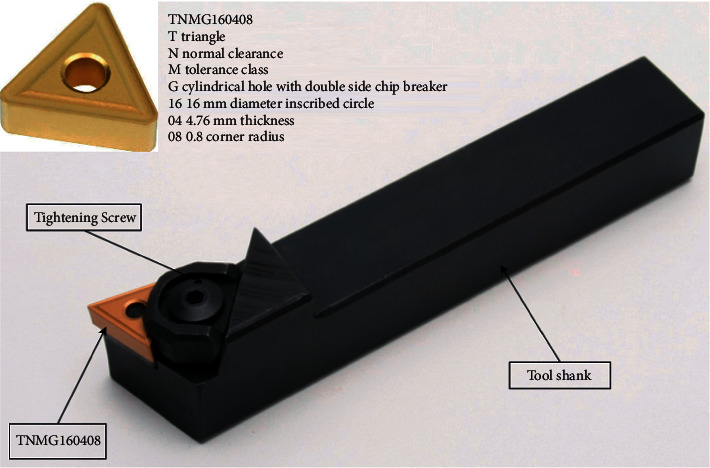
TNMG tool nomenclature.

**Figure 4:**
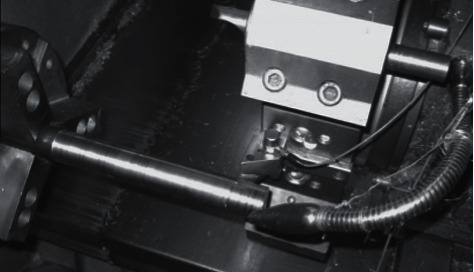
Accelerometer placement for tool condition monitoring.

The experimental setup utilized in the study comprises an ACE Micromatic–Classic 20T CNC turning machine, a signal conditioning unit, a piezoelectric accelerometer, and a personal computer to store the acquired signals. The single point carbide cutting tool as shown in Figure 3 was fixed onto the tool post. Workpiece made up of a mild steel shaft of 20 mm diameter was held by a pneumatic chuck. To acquire the vibration signals, the piezoelectric accelerometer (uniaxial) was fixed upon the tool holder using adhesive mounting technique. The signal conditioning unit (DACTRAN) was connected to the accelerometer in which the signals are conditioned and converted into digital form with the inbuilt analog to digital converter (ADC). The vibration signals post digital conversion is transferred and stored into a personal computer through a USB port.  Figure 4 represents the experimental setup used in this study.

### 2.2. Data Acquisition

Data acquisition (DAQ) is the process of creating digital values from the world around such that it can be visualized, analyzed, and stored in a computer. In this study, tool condition monitoring is carried out by acquiring vibration signals with the help of an accelerometer (piezo-electric sensor with sensitivity 10.26 mV/G). Accelerometers are capable of detecting both large and small vibrations. The working principle of the accelerometer states that output voltage magnitude is directly proportional to vibration signal intensity. The accelerometer was attached to the tool shank using adhesive techniques such that the vibration data are recorded for all conditions. A DACTRON FFT analyzer was used to convert the analog vibration signal into digital form. The acquired vibration data are further processed, and vibration plots are stored in a computer where transfer learning approach is applied to identify the best performing pretrained network for tool condition monitoring.

### 2.3. Simulation of Faults

The fault occurrences in the tool tip considered in this study were manually imparted based on the following procedure. Initially, a line (for reference) was drawn in parallel towards the tangent of the nose radius on a fresh tool tip. The distance between the highest nose point radius and the reference line was measured and stored. Then, the tool tip was introduced to a tool and cutter grinder machine in which the nose of the tool was machined for the measured distance. The bluntness of the tool was measured by calculating the difference between the distance from the reference line to the edge. The same procedure is repeated for achieving more pronounced bluntness and quantified. The various fault conditions simulated in the study are described as follows:

- Tool blunt low – Tool tip with a less blunt of 0.3 mm (tblent1)
- Tool blunt high – Tool tip with a more pronounced blunt of 0.6  mm (tblent2)
- Tool tip loose – Tool tip loosened by 1/12^th^ of a revolution of tightening screw (tiploose)

### 2.4. Experimental Procedure

An unused carbide tool tip (TNMG160408) was mounted on a tool holder attached to a tool post protruding from the turret head. Adhesive mounting technique was adopted in this study to mount the accelerometer on the tool holder. The parameters for signal acquisition, namely sampling length, sampling frequency, signal type etc., were fixed. The Nyquist sampling theorem states that the sampling frequency must be at least twice that of maximum frequency measured. Hence, the sampling frequency was chosen to be 24 kHz since the maximum frequency was found to be around 12 kHz. To start the process of turning, a mild steel rod of 20 mm diameter was fixed on the chuck (at live center) and rough turning was carried out to remove the top layer of the rod such that the surface is smoothened. The tool had a nose radius of 0.8 mm, and the cutting parameters represented in Table 2 were set on the CNC machine. Post turning on the cutting process, the DAQ system was turned on; and to avoid random variations in signal acquisition, the first few signals were dropped purposefully. The vibration signals were acquired from the accelerometer fixed on the tool holder after allowing the turning operation to stabilize for some time (about 1 min). A total of 100 signals were acquired after the stabilization of the turning operation. The parameters considered during the signal collection are depicted as follows:

- Sampling length: 8192 steps
- Sampling frequency: 24 kHz
- Number of instances for each condition: 100

## 3. Description of Convolutional Neural Networks (CNNs)

**Figure 5:**
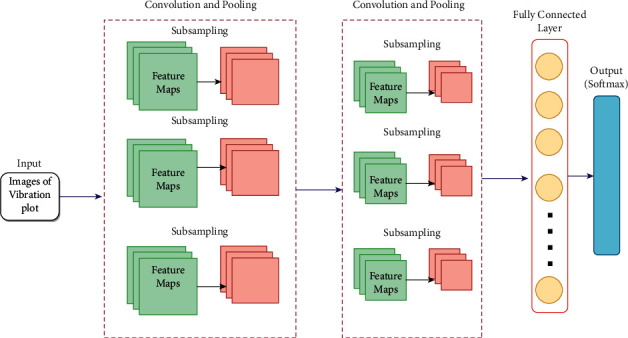
General architecture of convolutional neural networks.

CNN formulates a connection between the image and image features by creating a set of biases and weights. The architecture is designed in a hierarchical manner that works on an automatic feature learning algorithm. The classification ability of CNN is based on the features that were learnt during the process of convolutions. The CNN architecture is composed of sequential stacking of several layers, namely convolution layer, pooling layer, and fully connected layer. The general architecture of CNN architecture is provided in Figure 5.

The basic function of CNN is involved around the abovementioned layer groups, as given below:

- The input for a convolution layer is provided with the help of input layer which stores the image pixel values.
- The convolution layer is stacked next to the input layer that is filled with neurons of different weights and biases. The output of every neuron is calculated based on the product of volume and weights provided by the input. Rectified linear units (ReLU) is adopted widely as the activation function such that the nonlinearity among the problem is sustained.
- The immediate layer present next to convolution layer is the pooling layer which acts as the down sampling layer. Features of high dimension are sampled down to achieve the spatial dimensionality such that the number of parameters can be reduced.
- The fully connected layers complete the CNN architecture by providing the classification results for a particular problem. The classification is carried out by assigning a particular range of values to every class such that improved performance is achieved. Matrix form of image features are converted into vector form through the fully connected layers.

CNN is able to transform the original input layer by layer to generate class scores for classification and regression purposes using convolutional and down sampling techniques. It is therefore important to remember that it will not be enough to simply determine the overall design of CNN architecture. These models can take some time to build and refine. Now, let us analyze the individual layers in detail, describing their hyperparameters and connectivity.

### 3.1. Convolution Layer

The learning process of CNN is instrumented by the means of convolution layer. The collection of several learnable kernels or filters is dependent on the parameters assigned to the layers. The kernels function in such a way that they spread across the wide range of input by consuming lower spatial dimensionality. In the process of convolution, two-dimensional activation maps are created for every image fed into the filter throughout the spatial dimensionality. The scalar product of weights and volume is calculated for every image data point that passes through the kernel present in the convolution layer. The values created through each filter (generally termed as activations) triggers the network to learn significant features that are available in the spatial domain. The midpoint of the kernel is placed over the input vector through which the weighted sum of itself and any neighboring pixels are calculated and replaced. Also, the model complexity can be reduced by convolution layers by means of hyperparameter optimization. Depth (no. of filters), stride (movement of filter in one direction), and zero-padding (adding zeros around the border of input image) are the three hyperparameters that can optimize the performance of convolution layers.

### 3.2. Pooling Layer

Pooling layers are equipped in deep learning architectures for the purpose of dimensionality reduction of particular data. Adoption of pooling layers can reduce the computational complexity of the model by shrinking the number of parameters involved. Pooling layer acts upon every value of input activation map and utilizes the “MAX” function for dimensionality scaling. Pooling layers have a destructive nature and are classified into two forms depending upon their function, namely average and max pooling. Also, max pooling is the most widely used pooling layers due to the efficient performance on all data types. The size of the filter and stride length are commonly fixed at 2 × 2 to allow the pooling layer expand throughout the input spatial dimensionality.

### 3.3. Fully Connected Layer

The ultimate layer of any CNN network is composed of fully connected layers. The output from the final convolution or pooling layer acts as the input to the fully connected layer. The output of convolution or pooling layers will be a matrix that must be flattened before being fed into the fully connected layer. Sigmoid or softmax are adopted as the activation functions in the fully connected to perform the classification task for the given input data.

## 4. Condition Monitoring of Single Point Cutting Tool Using Pretrained Models

**Figure 6:**
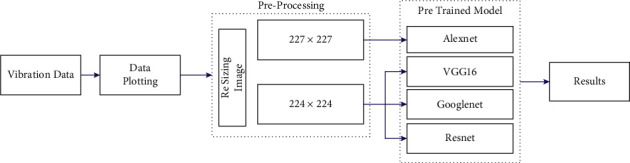
Overall workflow of tool condition monitoring using pretrained networks.

This section discusses about the various pretrained networks considered in the study to detect the condition of single point carbide cutting tool. Initially, the vibration signals were acquired for various conditions of the tool and stored in the form of vibration plots (images). The acquired images were further resized and preprocessed to a size of 227 × 227 or 224 × 224 according to the input requirement of the adopted pretrained model. Further, various renowned pretrained network models like VGG-16, AlexNet, ResNet-50, and GoogLeNet were utilized to perform image classification and identify the condition of the tool. Transfer learning was adopted in this study in which the initial weights of the networks trained on ImageNet are restored. Also, to apply the networks for custom dataset, the final output layers were replaced with new layers corresponding to the number of classes defined by the user.  Figure 6 represents the overall workflow of tool condition monitoring using pretrained networks. A brief description of the pretrained models considered in the study is provided as follows:

### 4.1. Dataset Formation and Preprocessing

**Figure 7:**
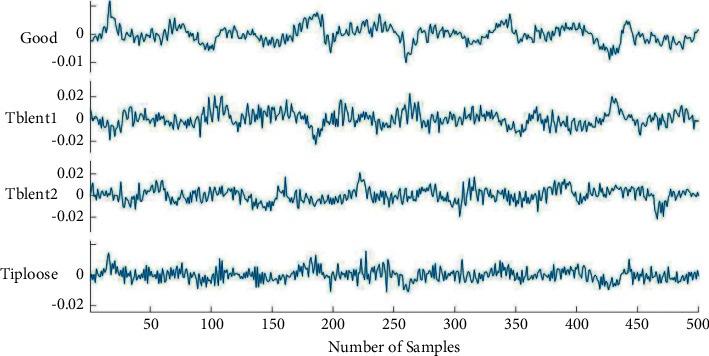
Vibration plots for tool tip unused and good (good), tool low blunt (tblent1), tool high blunt (tblent2), and tool tip loose (tiploose) conditions.

In this study, a dataset of images containing the conditions of the tool was created from the vibration signals acquired. Four test conditions, namely tool tip unused and good condition (good), tool low blunt (tblent1), tool high blunt (tblent2), and tool tip loose (tiploose) were considered. A total of 400 images (100 images for every class) were created using the acquired vibration signals. The acquired images were resized to a 224 × 224 or 227 × 227 such that the input size of the image is acceptable for the pretrained network adopted.  Figure 7 represents the vibration plots of various conditions of the tool collected for the study.

### 4.2. AlexNet Pretrained Network

AlexNet was introduced into the ImageNet competition that excelled as the first large-scale CNN architecture to perform classification. The network outperformed all state-of-the-art models (non-deep learning) by a substantial margin. AlexNet architecture resembles the LeNet architecture that is stacked with more deep layers. The network is stacked with 8 layers comprising of 61 million learnable parameters. The primary layers of the AlexNet are constituted by five convolution layers, two fully connected layer, and an output layer (also a fully connected layer). The AlexNet architecture was trained on ImageNet dataset with 1.2 million images representing 1000 object classes, with an input image size of 227 × 227 × 3. The first convolution layer of the AlexNet contains 11 × 11 filters in which 96 filters were applied at a stride of 4. The output of the first convolution layer produced a convolved image of size 55 × 55 × 96 carrying 35,000 parameters. The convolution layer is accompanied by a pooling layer with 3 filters of size 3 × 3 applied at stride of 2. The pooling layer returned an output volume of size 27 × 27 × 96 with no learnable parameters. No learning process is carried out in pooling layer since the layer is used as a down sampling layer to reduce dimensional complexity. Also, the learning process is confined to the weights of the neurons present in the convolutional layers. The process of convolution is continued further with the other convolution layers with varying filter sizes, namely 11 × 11, 5 × 5, and 3 × 3. Finally, a couple of fully connected layers of size 4096 converts the matrix form of images into vector form that are fed into the output layer. The layer (‘fc8) is made up of softmax activation function that performs the image classification. ReLU activation function is employed such that nonlinear problems can be addressed thereby making efficient decision.

### 4.3. VGG-16 Pretrained Network

The 2014 ImageNet competition saw innovations in architectures that displayed significant performance with more deeper layers. VGG-16 architecture proposed by Karen Simmonyan et al. is an architecture constructed with 16-layers. The VGG network was aimed at stacking more deeper network layers with smaller filter sizes such that effective convolution occurs. The name Virtual Geometry Group (VGG) was coined after a group of researchers from Oxford University. VGG network is much deeper than AlexNet and comes in two variants with 16 or 19 layers. The VGG network consists of very small filters of size 3 × 3 that are uniformly distributed among all the convolution layers to extract more distinct features. Periodic pooling was applied throughout the network layers such that the network structure becomes simple. The usage of smaller filter sizes reduces the number of learnable parameters thereby reducing the dimensional and computational complexity involved during convolution process. Smaller, deeper filters are used in VGG architecture rather than large, memory-consuming filters. The receptive field of the VGG architecture resembled the working of 7 × 7 convolution layers. VGG-16 architecture contains a combined total of 16 convolutional and fully connected layers.

### 4.4. GoogLeNet Pretrained Network

In the annual ILSVRC 2014, Szegedy et al. proposed a network architecture named GoogLeNet that was aimed at solving image classification and object detection. The architecture was composed of 22 layers that had its application extended into the fields of facial recognition, robotics, adversarial training, etc. The network is arranged with nine inception modules connected to four convolution, four max pooling, three average pooling, five fully connected, and three softmax layers. ReLU is the activation function adopted in fully connected layers that are supported by a dropout layer of 0.5 ratio. Inception modules present in GoogLeNet architecture enables the network to solve more complex computer vision problems. The identification of complex features through variations in convolution layer filter size is the prime advantage of adopting inception modules. Such process can help in reducing the computational time and dimensional complexity. Even though the architecture of GoogLeNet looks robust with 22 layers, the volume of trainable parameters is less in comparison with AlexNet.

### 4.5. ResNet Pretrained Network

Residual network (ResNet) was the most successful and efficient network in the annual ILSVRC 2015 developed by He et al. The major advantages of using ResNet architecture is high convergence rate with accurate classification. The common objects in context (COCO) dataset were used to train the ResNet architecture. Residual units were stacked together to form the ResNet architecture. ResNet architectures come in many forms depending on the number of residual units present and the variations in number of layers. ResNet architectures success was influenced by the application of identity shortcuts in which the value of output identity mimics the input values identity. Like other networks, ResNet is also composed of convolution pooling and fully connected layers. The ResNet architecture resembles VGG network architecture. However, the former is eight times deeper than the latter resulting in a greater number of learnable features. Overall, the ResNet-50 architecture considered in the study consists of 49 convolution layers and one fully connected layer.  Table 3 represents the various characteristic features of the adopted pretrained networks.

## 5. Results and Discussion

In this section, the performance of the pretrained models (AlexNet, VGG-16, GoogLeNet, and ResNet-50) for tool condition monitoring is evaluated. A total of four experiments were carried out based on variations in train-test split ratio, optimizer (or) solver, initial learning rate, and batch size. The overall experimentation was carried out in the desktop version of MATLAB 2019b using the deep learning toolbox and transfer learning package. The detailed experimental study is explained as follows:

### 5.1. Effect of Train-Test Split Ratio

**Figure 8:**
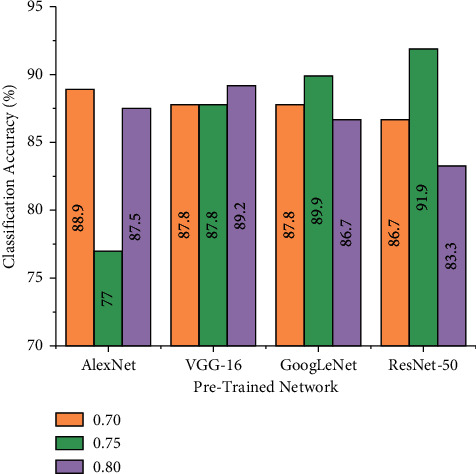
Performance of pretrained networks for varying train-test split ratio.

Train-test split ratio is the process of dividing the collected dataset into two subsets, namely training data and testing data. Training data are utilized to train your pretrained network, while the testing data are used to evaluate the trained model for the custom dataset. In this study, three train-test split ratios were experimented for all the pretrained networks by fixing default values for certain hyperparameters such that uniform evaluation is carried out. The hyperparameters such as solver (SGDM), batch size (10), and learning rate (0.0001) were kept to default values to identify the best train-test split ratio.  Figure 8 describes the performance of various pretrained networks for varying train-test split ratio.

From Figure 8, one can infer that the performance of each pretrained network varies with a change in the train-test split ratio. The observations made from Figure 8 state that AlexNet achieved a maximum accuracy of 88.90% for the train-test split ratios of 0.7. However, GoogLeNet and ResNet-50 achieve higher classification accuracy of 89.90% and 91.90% for train-test split ratio of 0.75, respectively. Additionally, one can observe that VGG-16 pretrained network achieved 89.20% classification accuracy for 0.80 train-test split ratio. Computing the overall accuracy of the pretrained networks, it can be observed that VGG-16 produces the best classification accuracy of 88.26%.

### 5.2. Effect of Optimizers

**Figure 9:**
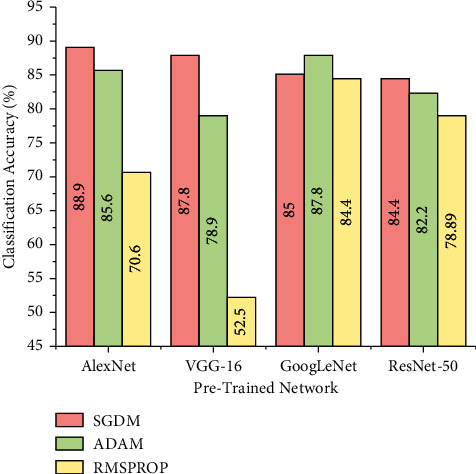
Performance of pretrained models for various solvers.

Optimizers or solvers are the algorithms that are adopted during training process such that the loss value is minimized to achieve an improved performance of the model. In this study, an experimentation was carried out by varying the solvers, namely stochastic gradient descent (sgdm), adaptive moment estimation (Adam), and root mean square propagation (RMSprop) to evaluate the performance of the model. The best performing train-test split ratio for each model was fixed based on the experimental results depicted in Section 5.1 that is, 0.7 for AlexNet, 0.75 for GoogLeNet and ResNet-50, and 0.80 for VGG-16.  Figure 9 depicts the performance of pretrained models for different solvers and load conditions.

Changing the optimizers can have an impact on the performance of the pretrained networks which is evident from Figure 9. The observations state that adoption of sgdm solver produced better classification accuracy for three pretrained networks, namely AlexNet with 88.90%, VGG-16 with 87.80%, and ResNet-50 with 84.40%. However, GoogLeNet achieved a maximum accuracy of 87.80% for Adam optimizer. VGG-16 displayed poor performance for RMSprop optimizer.

### 5.3. Effect of Learning Rate

**Figure 10:**
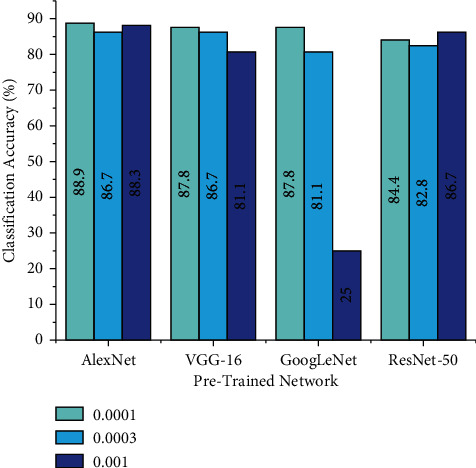
Performance of pretrained models for various learning rates.

Learning rate is one critical parameter that monitors the change in the training model with respect to the estimated error during every instance of model weight upgradation. Selecting the best learning rate can be challenging since small learning rate will elevate computational time, while higher learning rates will result in improper training. In this study, three values of learning weight like 0.001, 0.0001, and 0.0003 were used to evaluate the performance of the model. The other hyperparameters such as train-test split ratio and optimizer are fixed for the pretrained networks that are depicted as follows: AlexNet (0.7 train-test split, sgdm solver), VGG-16 (0.8 train-test split, sgdm solver), GoogLeNet (0.75 train-test split, Adam solver), and ResNet-50 (0.75 train-test split, sgdm solver).  Figure 10 depicts the performance of pretrained models for different learning rates.

From Figure 10, one can infer that each pretrained network performance varies with change in learning rate. The observations state that AlexNet, VGG-16, and GoogLeNet achieved a maximum accuracy of 88.90%, 87.80%, and 87.80%, respectively, for 0.0001 learning rate. However, ResNet-50 achieved a higher classification accuracy of 86.70% for 0.001 learning rate. Higher classification accuracy states that the model has learnt the features well and that the error value is minimal.

### 5.4. Effect of Batch Size

**Figure 11:**
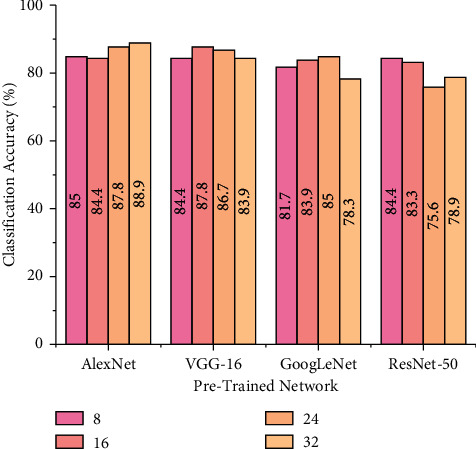
Performance of pretrained models for various batch sizes.

Batch size represents the number of samples that are propagated into a training network prior to model weight upgradation. The performance of the pretrained models in this study are evaluated for different batch sizes (8, 16, 24, and 32) with the selected best performing hyperparameters based on the experimental results mentioned in previous sections. The other hyperparameters like train-test split ratio, optimizer, and learning rate are fixed for the pretrained networks that are depicted as follows: AlexNet (0.7 train-test split, sgdm solver, 0.0001 learning rate), VGG-16 (0.8 train-test split, sgdm solver, 0.0001 learning rate), GoogLeNet (0.75 train-test split, Adam solver, 0.0001 learning rate), and ResNet-50 (0.75 train-test split, sgdm solver, 0.001 learning rate).  Figure 11 depicts the performance of pretrained models for different batch sizes and load conditions.

From Figure 11, one can observe that there are minimal changes incurred by changing the batch size. Every pretrained network displayed better classification accuracy for different batch sizes. For instance, AlexNet and VGG-16 produced higher classification accuracy of 88.90% and 87.80% for batch sizes 32 and 16, respectively. However, GoogLeNet and ResNet-50 produced better results of 85.00% and 84.40% for batch sizes 24 and 8, respectively. Among all the networks, AlexNet produced an overall accuracy of 86.52%. Increase in batch size will reduce the training time thereby leading to accelerated training progress. However, the generalization capability of the network reduces with higher batch sizes.

### 5.5. Comparative Study of Pretrained Models

**Figure 12:**
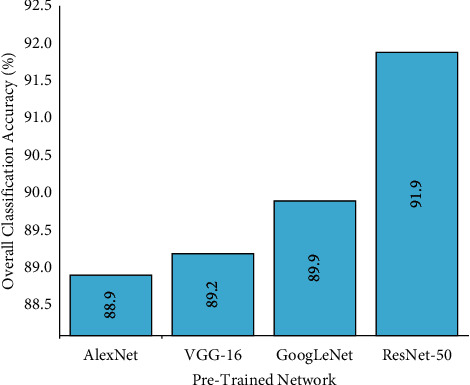
Performance comparison of pretrained models with optimal hyperparameters.

In this section, the performance evaluation of pretrained networks is discussed. Based on the experimental results obtained from previous sections, the optimal hyperparameters that enhance the performance of the pretrained models were identified. The list of best hyperparameters that produced an improved performance of pretrained models is provided in Table 4. The comparative study on the performance of pretrained networks with best hyperparameters is depicted in Figure 12. From Figure 12, one can infer that ResNet-50 established the utmost performance for the optimal hyperparameters selected. Based on the overall classification accuracy, superior classification and lower computational time consumed, one can suggest ResNet-50 for condition monitoring of single point carbide cutting tool. The training progress and confusion matrix of best performing networks are presented in Figures 7 and 8, respectively.

**Figure 13:**
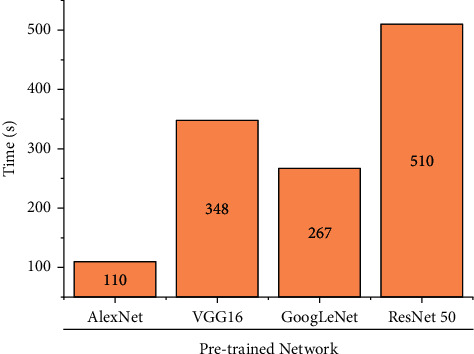
Computational time consumed by pretrained networks.

**Figure 14:**
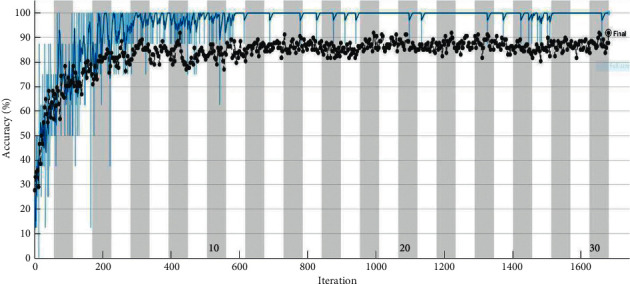
Training progress of ResNet-50 network for tool condition monitoring.

**Figure 15:**
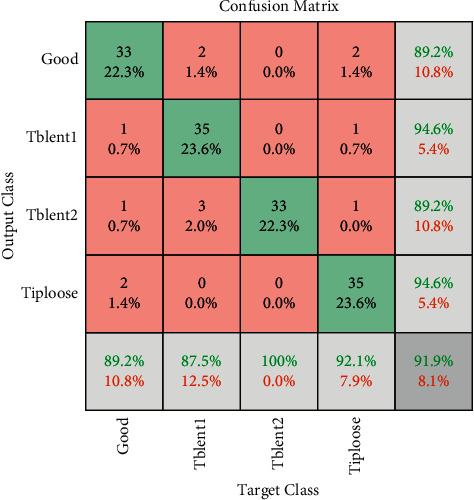
Confusion matrix of ResNet-50 network for tool condition monitoring.

Figure 13 represents the computational time consumed by every pretrained network. It can be observed that AlexNet being the simplest network consumes less computational time compared with complex ResNet-50 that consumed more computational time. From the training progresses represented in Figure 14, one can observe that the training process reaches saturation post 12 epochs. The sign of saturation represents that the ResNet-50 network is trained effectively for the given tool condition monitoring dataset. The overall loss during the training progress for all the networks have drastically reduced representing the selection of optimal hyperparameters. Additionally, Figure 15 depicts the confusion matrix of ResNet-50 architecture for tool condition monitoring. Confusion matrix and classification accuracy are used as the primary evaluation metrics in this study. In general, confusion matrix depicts the performance level of a particular model or algorithm. The evaluation of confusion matrix is carried out in regard to the instances classified in the major diagonal. The elements present in the major diagonal represent the correctly classified instances, while the other non-diagonal elements depict the misclassified instances. For instance, considering good condition, among 37 total instances, 33 instances were correctly classified as good condition. However, 4 instances were misclassified as tblent1 and tiploose. Misclassification can occur due to various reasons like noise interruption, poor signal quality, and similarity between acquired signals. Lesser number of misclassification instances infer that the network has learnt all the features effectively and that the loss during training is minimal. Thus, from the observation made, ResNet-50 is suggested as the best performing network for tool condition monitoring.

### 5.6. Comparative Study with State-of-the-Art Works

A comparative study is carried out in this section to display the superiority of the proposed technique over other state-of-the-art works presented in literature.  Table 5 displays the performance comparison of various techniques with the proposed technique. From Table 5, one can infer that the proposed method outclassed every other state-of-the-art works by displaying a classification accuracy of 91.9%. Support vector kernel functions displayed the second-best classification accuracy with 88.06% (v-SVC) and 87.50% (C-SVC).

## 6. Conclusion

In this study, four pretrained deep learning models, namely AlexNet, VGG-16, GoogLeNet, and ResNet-50, were applied to diagnose the condition of single-point carbide cutting tool from the vibration plots acquired Four test conditions, namely, tool tip unused and good condition (good), tool low blunt (tblent1), tool high blunt (tblent2), and tool tip loose (tiploose), were considered in the study. The pretrained networks are composed of CNN layers that perform an integrated approach of feature extraction, selection, and classification thereby formulating an end-to-end machine learning approach. The pretrained networks are capable of processing the vibration plots and providing accurate classification results. The experimental results enumerate that the adopted networks are capable of learning complex features and produce convincing classification results for monitoring the condition of the tool. Various hyperparameters like train-test split ratio, optimizer, learning rate, and batch size were altered, and the optimal hyperparameters were identified for all the networks. ResNet-50 was the best performing networks with 91.90% accuracy over AlexNet (88.90%), VGG-16 (89.20%), and GoogLeNet (89.90%). Based on the comparative studies, ResNet-50 is selected as the best performing network among the other networks considered in the study and is suggested for real-time application of condition monitoring of single point carbide cutting tool.

## Figures and Tables

**Table 1:** Related works on deep learning-based methods for mechanical systems.

Reference	Deep learning technique	Mechanical system
[31]	CNN with wavelet transform	Motor bearing
[32]	Hierarchical CNN	Roller bearing
[33]	CNN	
[27]	Deep belief network and sparse autoencoder	
[34]	Recurrent neural network	
[35]	Stacked autoencoder	Gear box
[36]	Generative adversarial network	
[37]	CNN	Centrifugal pump
[38]	Deep learning	Cutting tool
[39]	PCA and decision tree	
[40]	SVM	
[41]	FFBP, FFBPNN, and ANNBFIS	

**Table 2:** Machining parameters for cutting operation.

Cutting parameters	Parameter value
Depth of cut	0.5 mm
Cutting feed	0.1 mm/s
Spindle speed	600 rpm

**Table 3:** Characteristic features of adopted pretrained networks.

Model/network	Number of layers	Learnable parameters (in millions)	Input size of the image
AlexNet	8	60.0	227 × 227
VGG-16	16	137.0	224 × 224
GoogLeNet	22	7.1	224 × 224
ResNet-50	50	25.7	224 × 224

**Table 4:** Optimal hyperparameters for pretrained model.

Pretrained model	Hyperparameters			
	Split ratio	Optimizer	Learning rate	Batch size
AlexNet	0.70	SGDM	0.0001	32
VGG-16	0.80	SGDM	0.0001	16
GoogLeNet	0.75	ADAM	0.0001	24
ResNet-50	0.75	SGDM	0.001	8

**Table 5:** Performance comparison with state-of-the-art techniques.

Classifier	Classification accuracy (%)	Reference
Bayes Net	86.34	[42]
Naïve Bayes	85.28	
Decision tree	77.22	[39]
K-Star	78.00	[43]
v-SVC	88.06	[44]
C-SVC	87.50	
Proposed methodology	**91.90**	

The value depicted in bold represents the best classification accuracy produced in comparison with other state-of-the-art techniques. The proposed method delivers the highest classification accuracy than the other techniques investigated in the literature.

## Data Availability

The data used to support the findings of this study are included within the article. Further data or information can be obtained from the corresponding author upon request.
